# Modeling vaccination rollouts, SARS-CoV-2 variants and the requirement for non-pharmaceutical interventions in Italy

**DOI:** 10.1038/s41591-021-01334-5

**Published:** 2021-04-16

**Authors:** Giulia Giordano, Marta Colaneri, Alessandro Di Filippo, Franco Blanchini, Paolo Bolzern, Giuseppe De Nicolao, Paolo Sacchi, Patrizio Colaneri, Raffaele Bruno

**Affiliations:** 1 Department of Industrial Engineering, University of Trento, Trento, Italy; 2 Division of Infectious Diseases I, Fondazione IRCCS Policlinico San Matteo, Pavia, Italy; 3 Dipartimento di Scienze Matematiche, Informatiche e Fisiche, University of Udine, Udine, Italy; 4 Dipartimento di Elettronica, Informazione e Bioingegneria, Politecnico di Milano, Milan, Italy; 5 Department of Electrical, Computer and Biomedical Engineering, University of Pavia, Pavia, Italy; 6 IEIIT-CNR, Milan, Italy; 7 Department of Clinical, Surgical, Diagnostic, and Paediatric Sciences, University of Pavia, Pavia, Italy

**Keywords:** Epidemiology, Epidemiology

## Abstract

Despite progress in clinical care for patients with coronavirus disease 2019 (COVID-19)^1^, population-wide interventions are still crucial to manage the pandemic, which has been aggravated by the emergence of new, highly transmissible variants. In this study, we combined the SIDARTHE model^2^, which predicts the spread of SARS-CoV-2 infections, with a new data-based model that projects new cases onto casualties and healthcare system costs. Based on the Italian case study, we outline several scenarios: mass vaccination campaigns with different paces, different transmission rates due to new variants and different enforced countermeasures, including the alternation of opening and closure phases. Our results demonstrate that non-pharmaceutical interventions (NPIs) have a higher effect on the epidemic evolution than vaccination alone, advocating for the need to keep NPIs in place during the first phase of the vaccination campaign. Our model predicts that, from April 2021 to January 2022, in a scenario with no vaccine rollout and weak NPIs (${\cal{R}}_0$ = 1.27), as many as 298,000 deaths associated with COVID-19 could occur. However, fast vaccination rollouts could reduce mortality to as few as 51,000 deaths. Implementation of restrictive NPIs (${\cal{R}}_0$ = 0.9) could reduce COVID-19 deaths to 30,000 without vaccinating the population and to 18,000 with a fast rollout of vaccines. We also show that, if intermittent open–close strategies are adopted, implementing a closing phase first could reduce deaths (from 47,000 to 27,000 with slow vaccine rollout) and healthcare system costs, without substantive aggravation of socioeconomic losses.

## Abstract (web summary)

Integration of variables to account for different speeds of vaccination rollouts and transmission dynamics of new SARS-CoV-2 strains into the SIDARTHE-V model suggests that maintaining non-pharmaceutical interventions during the COVID-19 vaccination campaign in Italy is essential for minimizing mortality in Italy into 2022.

## Main

Since the severe acute respiratory syndrome coronavirus 2 (SARS-CoV-2) genome was sequenced^3^, researchers have rushed to develop vaccines to curb the spread of COVID-19 (refs. ^4,5^). Given the infeasibility of long-term lockdowns^6,7^ and of effective contact tracing at high case numbers, as well as the availability of several approved COVID-19 vaccines, many countries have invested in mass vaccination rollouts. As of 13 March 2021, four vaccines—Pfizer/BioNTech, Moderna, Oxford–AstraZeneca AZD1222 and J&J Ad26.COV2.S—have been approved by the European Medicines Agency and the Italian Medicines Agency. The reported efficacy rates are 94% and 95%, respectively, for the Moderna and Pfizer/BioNTech vaccines^8,9^, up to 81.3% for AZD1222 after the second dose with a longer prime–boost interval^10^ and up to 85% in preventing severe disease for J&J Ad26.COV2.S 28 d after vaccination^11^. All vaccines have been reported to have favorable safety profiles^8,9,11–14^. Italy’s vaccination program started in late December 2020^15,16^ and prioritized healthcare workers, nursing home residents and people over 80 years of age^17,18^. As of 26 March 2021, 2,787,749 people have been vaccinated in Italy with both doses (8,765,085 doses have been administered in total)^19^.

Multi-pronged countermeasures, including distancing, testing and tracing, are necessary to achieve a sustained reduction in infection cases^20^, even more so in light of the recent emergence of new SARS-CoV-2 variants^21^, such as B.1.1.7 and B.1.351, which are reported to have increased transmissibility^22,23^ and possibly cause more severe disease^24^ compared to the original strain. Vaccination alone is not expected to be able to control the spread of the infection, and a carefully planned vaccination campaign^25,26^ needs to be coordinated with continued implementation of NPIs^27^ until sufficient coverage is reached to make the case fatality rate (CFR) similar to that of seasonal influenza. Table 1 outlines the main findings and implications for policy of our study.

**Table 1:** Policy summary

**Background**	The second wave of the SARS-CoV-2 pandemic has severely affected Italy with a high CFR. Two potential game-changers now affect the evolution of the epidemic: the availability of vaccines and the emergence of more transmissible virus variants. We combine our compartmental epidemiological model with a new data-based model of healthcare costs to assess the effect of the vaccination campaign on the future evolution of the epidemic, in the presence of different NPIs and SARS-CoV-2 variants of concern.
**Main findings and limitations**	Even though mass vaccination has started, NPIs remain crucial to control the epidemic, in part owing to circulation of highly transmissible variants of SARS-CoV-2. Stricter restrictions curb transmission more than faster vaccine rollout. Easing NPIs leads to a surge of infection cases, calling for new closures, thus triggering intermittent restrictions. Pre-emptive strategies (first Close, then Open at low case numbers) could drastically reduce hospitalizations and deaths, without aggravating socioeconomic costs, with respect to a delayed intervention (first Open, then Close to prevent ICU saturation). As with any modeling study, there are inherent limitations. We think that our scenarios are outlined based on reasonable assumptions, but the actual epidemic evolution will depend on the adopted measures as well as the possible emergence of other variants.
**Policy implications**	Our findings strongly advocate for the need to keep NPIs in place in the first phase of the vaccination campaign until sufficient population immunity is reached. We also show the effectiveness of pre-emptive action: given a finite horizon and Close/Open phases of fixed length, closing first could spare tens of thousands of lives and reduce healthcare costs without aggravating socioeconomic losses by comparison with opening first.

**Fig. 1:**
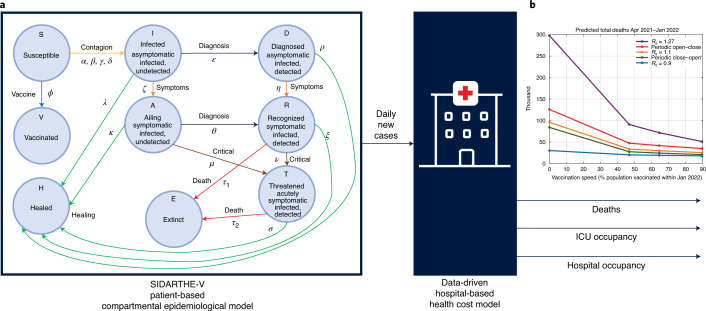
Model scheme and summary of our findings. **a**, Graphical scheme of our model (adapted from Giordano et al.^2^). The compartmental SIDARTHE-V epidemiological model provides daily new cases as an input to a novel data-based model of casualties and healthcare system costs. The SIDARTHE-V model captures the dynamic interactions among nine mutually exclusive infection stages in the population: S, Susceptible (uninfected); I, Infected (asymptomatic infected, undetected); D, Diagnosed (asymptomatic infected, detected); A, Ailing (symptomatic infected, undetected); R, Recognized (symptomatic infected, detected); T, Threatened (infected with life-threatening symptoms, detected); H, Healed (recovered); E, Extinct (dead); and V, Vaccinated (successfully immunized). The SIDARTHE-V model provides the time evolution of daily new infection cases, based on which the data-driven model computes the evolution of deaths and ICU and hospital occupancy. **b**, Death versus vaccination speed curves. For a given ${\cal{R}}_0$ profile, the curve gives the death toll in the period from April 2021 to January 2022 as a function of the average vaccination speed, measured as the fraction of vaccinated population at the end of the period. The death versus vaccination speed curves corresponding to a constant reproduction number are reported in purple (${\cal{R}}_0 = 1.27$), orange (${\cal{R}}_0 = 1.1$) and blue (${\cal{R}}_0 = 0.9$), whereas those corresponding to intermittent strategies are reported in red (Open–Close) and green (Close–Open).

With vaccines and variants as potential game-changers, new models to forecast epidemic scenarios and assess the associated healthcare costs are essential. Our proposed integrated model (Fig. 1a) uses the compartmental model SIDARTHE^2^ (which we have extended here to include the effects of vaccination and now termed SIDARTHE-V) to provide the predicted evolution of new positive cases; based on new positive cases, a new data-based dynamic model derived from Italian field data computes the time profile of the resulting healthcare system costs, including hospital and intensive care unit (ICU) occupancy and deaths. Although age classes are not explicitly included in our compartmental model, they are accounted for by the data-driven model. To capture the progressive vaccination in reverse age order, the model takes into account age-dependent aggravation and death probability (Extended Data Fig. 5). Details are provided in the Methods.

We compare different scenarios to assess the effect of mass vaccination campaigns with different paces, in the presence of varying profiles of the reproduction number ${\cal{R}}_0$ over time, due to specific SARS-CoV-2 variants and/or restrictions. We consider four effective vaccination schedules (Extended Data Fig. 4), obtained by modulating linearly the speed of the four phases, T1–T4, of Italy’s vaccination plan^28^ and yielding a different fraction of immunized people within January 2022: absent (0%), slow (47%), medium (64%) and fast (90%). We also consider five different time profiles of ${\cal{R}}_0$: constant ${\cal{R}}_0 = 1.27$ (high transmission); Open–Close periodic ${\cal{R}}_0$ with average value of 1.1, in which 1-month Openings (${\cal{R}}_0 = 1.27$: leaving schools and shops open, wearing face masks and keeping physical distance) alternate with 1-month Closures (${\cal{R}}_0 = 0.9$: closing schools, shops, restaurants and entertainment places), starting with an Opening phase; constant ${\cal{R}}_0 = 1.1$; Close–Open periodic ${\cal{R}}_0$ with average value of 1.1, in which 1-month Closures alternate with 1-month Openings, starting with a Closure phase; constant ${\cal{R}}_0 = 0.9$ (eradication).

Our main findings are summarized by the deaths versus speed curves in Fig. 1b, which show mortality as a function of the vaccination rollout speed for each ${\cal{R}}_0$ profile. Vaccination is assumed to reduce viral transmission as well as disease severity and risk of death. The different vaccination schedules could also be interpreted as different proportions of infections, diseases and deaths that the vaccine successfully stops, thus constituting a sensitivity analysis. The combination of the four vaccination schedules with the five ${\cal{R}}_0$ profiles leads to 20 distinct scenarios (Fig. 1b). Eradication is associated with an almost constant curve; however, with ${\cal{R}}_0 = 1.27$, the proportion of deaths with slow, medium and fast vaccination schedules could be as small as 30%, 24% and 17% of the 298,000 deaths with no vaccination, respectively. The deaths versus speed curves are flatter when ${\cal{R}}_0$ is kept smaller; implementation of stringent NPIs drastically reduces sensitivity to vaccination delays. Restrictive containment strategies (${\cal{R}}_0 = 0.9$) lead to a number of deaths that could be as small as 10% of deaths with weak restrictions (${\cal{R}}_0 = 1.27$): depending on the ${\cal{R}}_0$ profile, deaths in the period from April 2021 to January 2022 vary in the range of 30,000–298,000 (no vaccination rollout), 20,000–91,000 (slow vaccination rollout), 19,000–72,000 (medium vaccination rollout) and 18,000–51,000 (fast vaccination rollout). Therefore, NPIs appear to have a stronger effect on mortality than vaccination speed. When planning mid-term interventions, pre-emption reduces mortality and healthcare system costs at no additional socioeconomic cost by comparison with delayed implementation. Both intermittent restrictions with the same average ${\cal{R}}_0$ involve similar socioeconomic costs, but starting with a Closing phase improves on constant containment, which is better than starting with an Opening phase. For all vaccination schedules, the Close–Open strategy saves more than 14,000 lives compared to Open–Close. Hospital and ICU occupancy as a function of the vaccination speed follow a similar pattern (Extended Data Fig. 8).

**Fig. 2:**
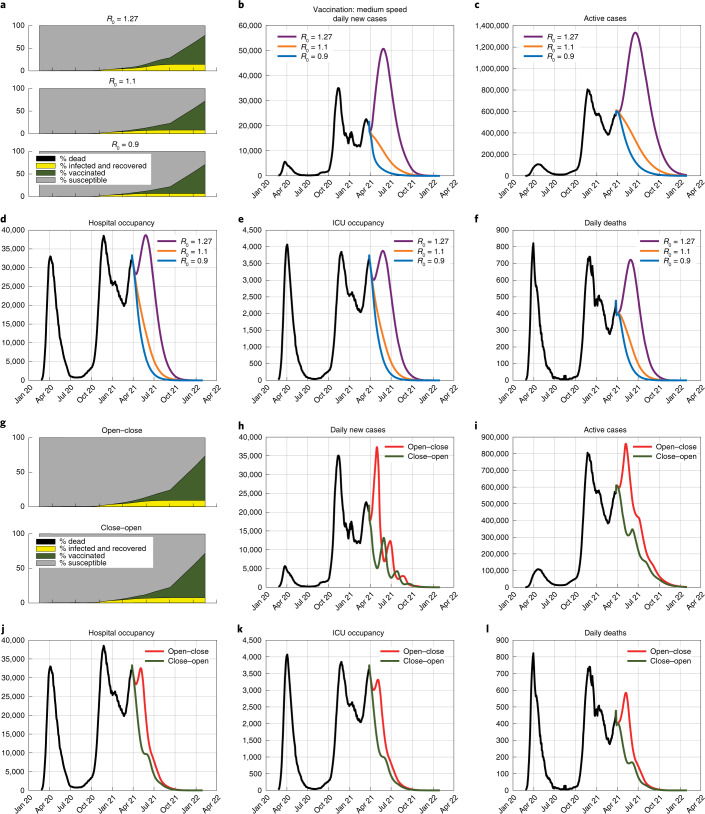
The effect of different constant values of ${\mathbf{{\cal{R}}}}_0$ and intermittent strategies. Time evolution of the epidemic, in the presence of medium-speed vaccination (64% of the population vaccinated within January 2022), when: **a**–**f**, different constant values of ${\cal{R}}_0$, namely ${\cal{R}}_0 = 1.27$ (purple), ${\cal{R}}_0 = 1.1$ (orange) and ${\cal{R}}_0 = 0.9$ (blue), are assumed, resulting from different variants and/or containment strategies, and when: **g**–**l**, two alternative intermittent strategies are enforced, with an average value of ${\cal{R}}_0$ equal to 1.1. The Open–Close strategy (red) switches every month between ${\cal{R}}_0 = 1.27$ and ${\cal{R}}_0 = 0.9$ starting with ${\cal{R}}_0 = 1.27$. The Close–Open strategy (green) switches every month between ${\cal{R}}_0 = 0.9$ and ${\cal{R}}_0 = 1.27$ starting with ${\cal{R}}_0 = 0.9$. **a**,**g**, Time evolution of the fractions of susceptibles, vaccinated, infected and recovered, and dead. **b**,**h**, Daily new cases. **c**,**i**, Active cases. **d**,**j**, Hospital occupancy. **e**,**k**, ICU occupancy. **f**,**l**, Daily deaths.

Considering a medium vaccination speed, Fig. 2a–f shows the epidemic evolution for different constant values of ${\cal{R}}_0$ (the scenarios in the absence of vaccination are in Extended Data Fig. 2). Despite vaccination and implementation of current containment measures, a higher transmissibility due to the spread of new variants would cause a dramatic surge in infection cases, leading, within 2 months, to a peak of almost 4,000 ICU beds needed and more than 700 daily deaths. To prevent this from happening and to reduce hospital occupancy and mortality, ${\cal{R}}_0$ can be reduced through increased stringency of NPIs, particularly in the presence of highly transmissible variants.

The need to implement new restrictions is likely to trigger intermittent containment measures, with the alternation of higher-${\cal{R}}_0$ and lower-${\cal{R}}_0$ phases^29,30^. In Open–Close strategies, closures are delayed and applied only in anticipation of the pressure on the healthcare system becoming unbearable. Each intermittent Open–Close strategy can be associated with a Close–Open strategy that alternates opening and closing phases of the same duration, with the only difference of starting with a closure. Figure 2g–l compares the two different intermittent strategies, with average ${\cal{R}}_0$ equal to 1.1, under medium-speed vaccination (the scenarios in the absence of vaccination are in Extended Data Fig. 3). Opening first (Open–Close) or closing first (Close–Open) strongly affects healthcare system costs (which depend on case numbers), whereas socioeconomic costs (which depend on the duration and stringency of restrictions) are substantially unchanged. Without aggravation of social and economic losses with respect to an Open–Close strategy, a pre-emptive Close–Open strategy drastically reduces forthcoming infection numbers (decreasing the peak of daily new cases from 38,000 to 14,000), hospital and ICU occupancy and deaths (decreasing the peak of daily deaths from 600 to 400). Even though the average ${\cal{R}}_0$ is above 1, the effective reproduction number ${\cal{R}}_t = {\cal{R}}_0S(t)$ goes below 1 due to the decreasing susceptible fraction *S*(*t*); hence, the epidemic is eventually suppressed (Methods).

**Fig. 3:**
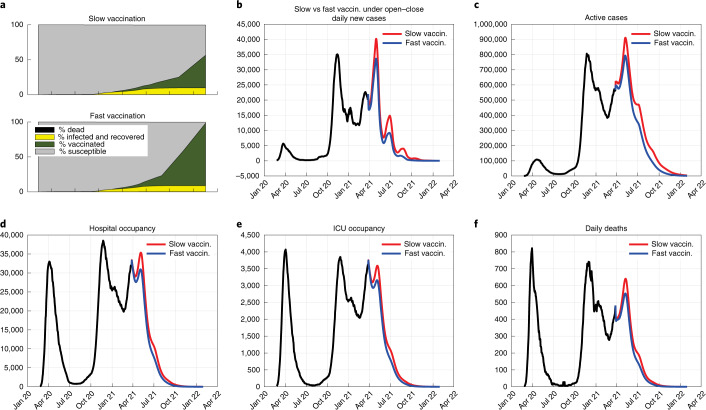
The effect of different vaccination paces. Time evolution of the epidemic, with an intermittent Open–Close strategy enforced, in the presence of slow vaccination (47% of the population vaccinated within January 2022, red) or fast vaccination (90% of the population vaccinated within January 2022, blue). **a**, Time evolution of the fractions of susceptible, vaccinated, infected and recovered, and dead. **b**, Daily new cases. **c**, Active cases. **d**, Hospital occupancy. **e**, ICU occupancy. **f**, Daily deaths.

Finally, we comparatively assess the effect of mass vaccination with different paces (which could be also interpreted as the effect of different vaccine efficacy). We assume that the number of reinfections occurring within the considered horizon is negligible. Figure 3 compares the effect of slow versus fast vaccination under the intermittent Open–Close strategy. Although vaccination leads to a net reduction in deaths and hospital and ICU occupancy compared to the corresponding scenario without vaccination, the difference in effect between slow and fast vaccination is modest. The speed of vaccination becomes more important with a higher ${\cal{R}}_0$, at the price of many more deaths. In Extended Data Figs. 6 and 7, we also consider an adaptive vaccination scenario, where an increase in the number of current infection cases leads to a reduction of the vaccination rate, due to the augmented strain on the healthcare system. Both mortality and healthcare system costs increase, reinforcing conclusions about the greater importance of containment measures over vaccination rates.

There are limitations to our study. The SIDARTHE-V model is a mean-field compartmental model, which relies on the assumption of a large population with homogeneous mixing and provides predictions that are averaged over the whole population; hence, geographical heterogeneity is not taken into account. More complex and detailed models, which account for spatial effects, social networks and the specificity of individual behaviors, can be developed and used to evaluate vaccination strategies^31^. Also, we assumed that vaccination is effective against SARS-CoV-2 variants. However, several concerns are raised regarding variants and their potential for vaccine-induced immunity escape^32,33^; preliminary reports suggest that some COVID-19 vaccines might retain efficacy against variants^34,35^, although it might be attenuated^36^, whereas data suggest that Oxford–AstraZeneca AZD1222 might be less effective against B.1.351 (ref. ^37^). In our scenarios, we also optimistically assumed that successfully vaccinated individuals gain protection against death and hospitalization starting 3 weeks after the first vaccine dose rather than after the second dose.

Vaccination started with slow rates, and priority was given to healthcare personnel, thus delaying the CFR decrease: even under the fast rollout, the CFR would not be halved before June (Extended Data Fig. 4c). Because the decrease rate of the CFR cannot be made arbitrarily fast, due to availability and administration rate of vaccines, our findings confirm that, in the first phase of the mass vaccination campaign, NPIs are crucial, regardless of the (realistic) vaccination speed. Given the circulation of highly transmissible SARS-CoV-2 variants and the risk of potential emergence of vaccine-resistant mutations, ${\cal{R}}_0$ must be kept low until a sufficient level of population immunity is achieved and a large enough portion of the vulnerable population has been immunized. Only then can NPIs be safely and gradually released; the time at which this happens will depend on the speed of vaccine rollout.

To outrun the faster spread of the virus variants, the United Kingdom (UK) launched an extensive COVID-19 vaccination campaign, accelerated by extending the interval between doses: more than 29 million people have received at least one vaccine dose as of 25 March, which has reduced deaths and hospital admissions^38^. Our model confirms that implementation of strong NPIs could bring the epidemic under control without vaccines or before reaching population immunity, as happened in the UK during January 2021: the highly transmissible B.1.1.7 variant, which first emerged in Kent, UK, and spread throughout the UK, was brought under control by lockdown restrictions kept in place during the first crucial phases of the vaccine rollout campaign. In the meantime, vaccine rollout in the UK has enabled planning subsequent gradual release of NPIs^38,39^. Although the UK is heading into the second phase of the vaccination campaign, Italy is at an early phase, close to the UK’s first. To contain the new Italian outbreak of SARS-CoV-2, driven by the new variants of concern, it is important to maintain NPIs to prevent an uncontrolled surge in the number of infections, hospitalizations and deaths, because vaccination alone will be insufficient to control the epidemic. In parallel, accelerating the vaccination campaign, as was done in the UK (perhaps also by increasing the interval between doses), would be worth considering.

## Methods

Our overall model (Fig. 1a) combines the flexibility and insight of compartmental models with the intrinsic robustness of a black-box healthcare system cost model based on observed data. The SIDARTHE-V model, including the compartment of vaccinated individuals (first block in Fig. 1a), generates the predicted evolution of new positive cases, which is used by the data-based model (second block in Fig. 1a) that captures hospitalization flows and quantifies healthcare system costs in terms of deaths and of hospital and ICU occupancy.

The data used to inform the model are taken from publicly available repositories and reports: https://github.com/pcm-dpc/COVID-19/tree/master/dati-andamento-nazionale for epidemiological data on the evolution of the COVID-19 epidemic in Italy until 12 March 2021; https://www.epicentro.iss.it/coronavirus/bollettino/Bollettino-sorveglianza-integrata-COVID-19_13-gennaio-2021.pdf for age-dependent CFRs; and http://dati.istat.it/Index.aspx for demographic information on the Italian population (needed to take age classes into account). Indeed, although age classes are not explicitly included in our compartmental SIDARTHE-V model, they are taken into account by the data-based model for healthcare system costs, which quantifies hospitalizations, ICU occupancy and deaths. The CFRs (and hospitalization rates) are computed by taking population age classes into account, as shown in Extended Data Fig. 5, using demographic information from http://dati.istat.it/Index.aspx.

### SIDARTHE-V compartmental model

The SIDARTHE-V compartmental model shown in Fig. 1a extends the SIDARTHE model, introduced by Giordano et al.^2^, by including the effect of vaccination. This leads to nine possible stages of infection: susceptible individuals (S) are uninfected and not immunized; infected individuals (I) are asymptomatic and undetected; diagnosed individuals (D) are asymptomatic but detected; ailing individuals (A) are symptomatic but undetected; recognized individuals (R) are symptomatic and detected; threatened individuals (T) have acute life-threatening symptoms and are detected; healed individuals (H) have had the infection and recovered; extinct individuals (E) died because of the infection; and vaccinated individuals (V) have successfully obtained immunity without having been infected.

The dynamic interaction among these nine clusters of the population is described by the following nine ordinary differential equations, describing how the fraction of the population in each cluster evolves over time:

1 $$\dot S\left( t \right) = - S\left( t \right)\left( {\alpha I\left( t \right) + \beta D\left( t \right) + \gamma A\left( t \right) + \delta R\left( t \right)} \right) - \varphi (S(t))$$

2 $$\dot I\left( t \right) = S\left( t \right)\left( {\alpha I\left( t \right) + \beta D\left( t \right) + \gamma A\left( t \right) + \delta R\left( t \right)} \right) - \left( {\varepsilon + \zeta + \lambda } \right)I\left( t \right)$$

3 $$\dot D\left( t \right) = \varepsilon I\left( t \right) - \left( {\eta + \rho } \right)D\left( t \right)$$

4 $$\dot A\left( t \right) = \zeta I\left( t \right) - \left( {\theta + \mu + \kappa } \right)A\left( t \right)$$

5 $$\dot R\left( t \right) = \eta D\left( t \right) + \theta A\left( t \right) - \left( {\nu + \xi + \tau _1} \right)R\left( t \right)$$

6 $$\dot T\left( t \right) = \mu A\left( t \right) + \nu R\left( t \right) - \left( {\sigma + \tau _2} \right)T\left( t \right)$$

7 $$\dot H\left( t \right) = \lambda I\left( t \right) + \rho D\left( t \right) + \kappa A\left( t \right) + \xi R\left( t \right) + \sigma T\left( t \right)$$

8 $$\dot E\left( t \right) = \tau _1R(t) + \tau _2T\left( t \right)$$

9 $$\dot V\left( t \right) = \varphi (S(t))$$

The uppercase Latin letters (state variables) represent the fraction of population in each stage, whereas all the considered parameters, denoted by Greek letters, are positive numbers and have the following meaning:

- The contagion parameters *α*, *β*, *γ* and *δ*, respectively, denote the transmission rate (defined as the probability of disease transmission in a single contact multiplied by the average number of contacts per person) due to contacts between a Susceptible individual and an Infected, a Diagnosed, an Ailing or a Recognized individual. These parameters can be modified by social distancing policies (for example, closing schools, remote working and lockdown) as well as physical distancing, adoption of proper hygiene behaviors and use of personal protective equipment. The risk of contagion due to Threatened individuals, treated in proper ICUs, is assumed to be negligible.
- The diagnosis parameters *ε* and *θ*, respectively, denote the probability rate of detection, relative to asymptomatic and symptomatic cases. These parameters, also modifiable, reflect the level of attention on the disease and the number of tests performed over the population: they can be increased by enforcing a massive contact tracing and testing campaign.
- The symptom onset parameters *ζ* and *η* represent the probability rate at which an infected individual, respectively, undetected and detected, develops clinically relevant symptoms. Although disease dependent, they might be partially reduced by improved therapies and acquisition of immunity against the virus.
- The critical/aggravation parameters *μ* and *v*, respectively, denote the rate at which undetected and detected infected symptomatic individuals develop life-threatening symptoms. The parameters can be reduced by means of improved therapies and acquisition of immunity against the virus.
- The mortality parameters *τ*_1_ and *τ*_2_, respectively, denote the mortality rate for infected individuals with symptoms (presumably in hospital wards) and with acute symptoms (presumably in ICUs) and can be reduced by means of improved therapies.
- The healing parameters *λ*, *κ*, *ξ*, *ρ* and *σ* denote the rate of recovery for the five classes of infected individuals and can be increased thanks to improved treatments and acquisition of immunity against the virus.
- The vaccination function *φ*(*S*(*t*)) represents the rate at which susceptible individuals successfully achieve immunity through vaccination (the rate depends on both the actual vaccination rate and the vaccine efficacy); possible choices are the state-dependent *φ*(*S*(*t*)) = *φS*(*t*), leading to an exponential decay of the number of susceptible individuals, and *φ*(*S*(*t*)) = *φS*(*t*) > 0 as long as *S*(*t*) > 0 (*φ*(*S*(*t*) = 0 otherwise), leading to a linear decay. In the latter case, *φ*(*t*) can be piecewise constant, as in the vaccination profiles in Extended Data Fig. 4. It is worth stressing that any vaccine (Pfizer/BioNTech, Moderna, Oxford–AstraZeneca, J&J and any other) can be considered within the model as the inducer of immunity, without altering the model validity.

Concerning the duration of immunity, correlates of protection against SARS-CoV-2 infection in humans are not yet established, but the results of a clinical trial with the mRNA-1273 vaccine show that, despite a slight expected decline in titers of binding and neutralizing antibodies, mRNA-1273 has the potential to provide durable humoral immunity: as the natural infection, which produces variable antibody longevity and might induce robust memory B cell responses, also mRNA-1273 vaccine elicited primary CD4 type 1 helper T responses 43 d after the first vaccination, and protection persists after 119 d^40^. Although it is unclear how long protective effects last beyond the first few months after vaccination, some studies suggest that the elicited neutralizing activity is maintained for up to 8 months after the natural infection with SARS-CoV-2 (refs. ^41,42^). Reasonably, considering that a similar pattern of responses lasting over time will also emerge after vaccinations, it would be at least unlikely that any potential reinfection would occur over the horizon considered by our scenarios. This is why reinfections have not been explicitly considered in the model, given that we are focused on short-term horizons.

Also, it is worth stressing that we are considering the rate of successful immunization, not of vaccine dose administration (this is why immunity is built up with a slower pace with respect to the expected vaccine roll-out logistics); people who get vaccinated, but for whom the vaccine is not effective, remain susceptible and are, therefore, equally at risk of serious disease and death.

The system is compartmental and has the mass conservation property: as it can be immediately checked, $\dot S\left( t \right) + \dot I\left( t \right) + \dot D\left( t \right) + \dot A\left( t \right) + \dot R\left( t \right) + \dot T\left( t \right) + \dot H\left( t \right) + \dot E\left( {\mathrm{t}} \right) + \dot V({\mathrm{t}}) = 0$, hence the sum of the states (total population) is constant. Because the variables denote population fractions, we have:

$$S(t) + I(t) + D(t) + A(t) + R(t) + T(t) + H(t) + E(t) + V(t) = 1,$$

where 1 denotes the total population, including deceased. Note that *H*(*t*), *E*(*t*) and *V*(*t*) are cumulative variables that depend only on the other ones and on their own initial conditions.

Given an initial condition *S*(0), *I*(0), *D*(0), *A*(0), *R*(0), *T*(0), *H*(0), *E*(0) and *V*(0) summing up to 1, if the vaccination function *φ*(*S*(*t*)) > 0 as long as *S*(*t*) > 0, the variables converge to

$$\bar S = 0,\bar I = 0,\bar D = 0,\bar A = 0,\bar R = 0,\bar T = 0,\bar H \ge 0,\bar E \ge 0,\bar V \ge 0,$$

with $\bar H + \bar E + \bar V = 1$. So only the vaccinated/immunized, the healed and the deceased populations are eventually present, meaning that the epidemic phenomenon is over. All the possible equilibria are given by $\left( {0,0,0,0,0,0,\bar H,\bar E,\bar V} \right)$, with $\bar H + \bar E + \bar V = 1$.

To better understand the system behavior, we partition it into three subsystems: the first includes just variable *S* (corresponding to susceptible individuals); the second, which we denote as the *IDART* subsystem, includes *I*, *D*, *A*, *R* and *T* (the infected individuals); and the third includes variables *H*, *E* and *V* (representing healed, defunct and vaccinated/immunized).

The overall system can be seen as a positive linear system subject to a feedback signal *u*. Defining *x* = [*I D A R T*]^T^, we can rewrite the *IDART* subsystem as

10 $$\dot x\left( t \right) = Fx\left( t \right) + bu\left( t \right) = \left[ {\begin{array}{*{20}{l}} { - r_1} \hfill & 0 \hfill & 0 \hfill & 0 \hfill & 0 \hfill \\ \varepsilon \hfill & { - r_2} \hfill & 0 \hfill & 0 \hfill & 0 \hfill \\ \zeta \hfill & 0 \hfill & { - r_3} \hfill & 0 \hfill & 0 \hfill \\ 0 \hfill & \eta \hfill & \theta \hfill & { - r_4} \hfill & 0 \hfill \\ 0 \hfill & 0 \hfill & \mu \hfill & \nu \hfill & { - r_5} \hfill \end{array}} \right]x(t) + \left[ {\begin{array}{*{20}{l}} 1 \hfill \\ 0 \hfill \\ 0 \hfill \\ 0 \hfill \\ 0 \hfill \end{array}} \right]u(t)$$

11 $$y_S\left( t \right) = c^ \top x\left( t \right) = \left[ {\begin{array}{*{20}{c}} \alpha & \beta & \gamma & \delta & 0 \end{array}} \right]x(t)$$

12 $$y_H\left( t \right) = f^ \top x\left( t \right) = \left[ {\begin{array}{*{20}{c}} \lambda & \rho & \kappa & \xi & \sigma \end{array}} \right]x(t)$$

13 $$y_E\left( t \right) = d^ \top x\left( t \right) = \left[ {\begin{array}{*{20}{c}} 0 & 0 & 0 & {\tau _1} & {\tau _2} \end{array}} \right]x(t)$$

14 $$u\left( t \right) = S(t)y_S\left( t \right)$$

where $r_1 = \varepsilon + \zeta + \lambda$, $r_2 = {\upeta} + \rho$, $r_3 = {\uptheta} + {\upmu} + \kappa$, $r_4 = {\upnu} + \xi + \tau _1$ and $r_5 = {\upsigma} + \tau _2$. The remaining variables satisfy the differential equations

15 $$\dot S\left( t \right) = - S\left( t \right)y_S\left( t \right) - \varphi \left( {S\left( t \right)} \right)$$

16 $$\dot H(t) = y_H(t)$$

17 $$\dot E(t) = y_E(t)$$

18 $$\dot V(t) = \varphi \left( {S\left( t \right)} \right)$$

We can also distinguish between diagnosed healed *H*_*D*_(*t*), evolving as

$$\dot H_D\left( t \right) = \rho D\left( t \right) + \xi R\left( t \right) + \sigma T\left( t \right),$$

and undiagnosed healed *H*_*U*_(*t*), evolving as

$$\dot H_U\left( t \right) = \lambda I\left( t \right) + \kappa A\left( t \right).$$

Then, the overall system is described by the infection stage dynamics

$$\dot x\left( t \right) = (F + bS\left( t \right)c^ \top )x\left( t \right)$$

along with the equations for *S*(*t*), *E*(*t*) and *H*(*t*) (or, equivalently, *H*_*D*_(*t*) and *H*_*U*_(*t*)).

The parametric reproduction number ${\cal{R}}_0$ is the *H*_*∞*_ norm of the positive system from *u* to *y*_*s*_ with parameters tuned at the beginning of the epidemic—that is, when the fraction of susceptible individuals is 1. A simple computation leads to

$${\cal{R}}_0 = \frac{{\alpha + \frac{{\beta \varepsilon }}{{r_2}} + \frac{{\gamma \zeta }}{{r_3}} + \delta (\frac{{\eta \varepsilon }}{{r_2r_4}} + \frac{{\zeta \theta }}{{r_3r_4}})}}{{r_1}}$$

All the parameters but *φ* are represented in ${\cal{R}}_0$. Because these parameters depend on the adopted containment measures, on the effectiveness of therapies and of the efficacy of testing and contact tracing, ${\cal{R}}_0$ is time varying in principle. Conversely, the basic reproduction number is the value of the parametric reproduction number at the first onset of the epidemic outbreak, and its value was estimated to range between 2.43 and 3.1 for SARS-CoV-2 in Italy^43^. The current reproduction number ${\cal{R}}_t$ is the product between ${\cal{R}}_0$ and the susceptible fraction: ${\cal{R}}_t = {\cal{R}}_0S(t)$. Notice that ${\cal{R}}_0$ depends linearly on the contagion parameters. A thorough parameter sensitivity analysis has been worked out for the SIDARTHE model^2^.

Fundamental mathematical results on the stability and convergence of the model in the absence of vaccination (that is, *φ*(*S*(*t*)) = 0) are summarized next.

The system (10), (14) with constant parameters and constant susceptible population $S\left( {\bar t} \right)$ is asymptotically stable if and only if ${\cal{R}}_{\bar t} < 1$. The equilibrium point $\bar x,\bar S$ of system (10), (14) with constant parameters after $\bar t$ is given by $\bar x = 0$ and $\bar S$ satisfying

$${\mathrm{ln}}\left( {\frac{{S(\bar t)}}{{\bar S}}} \right) + {\cal{R}}_0\left( {\bar S - S\left( {\bar t} \right)} \right) = - c^ \top F^{ - 1}x(\bar t)$$

The condition ${\cal{R}}_t = {\cal{R}}_0S\left( t \right) < 1$ is always verified after a certain time instant, so that $x\left( t \right) \to 0$ and $S\left( t \right) \to \bar S$ with ${\cal{R}}_0\bar S < 1$.

As a consequence, epidemic suppression is achieved when the inequality ${\cal{R}}_t = {\cal{R}}_0S\left( t \right) < 1$ is always verified from a certain moment onward.

### Fit of the SIDARTHE-V model for the COVID-19 epidemic in Italy

We infer the parameters for models (1)–(9) based on the official data (source: Protezione Civile and Ministero della Salute) about the evolution of the epidemic in Italy from 24 February 2020 through 26 March 2021. We turn the data into fractions over the whole Italian population (~60 million) and adopt a best-fit approach to find the parameters that locally minimize the sum of the squares of the errors.

With parameters estimated based on data until 26 March 2021, the SIDARTHE-V model reproduces the second wave of infection and feeds the health cost model that quantifies the healthcare system costs in different scenarios, encompassing Close–Open strategies during the mass vaccination campaign.

**Extended Data Fig. 1:**
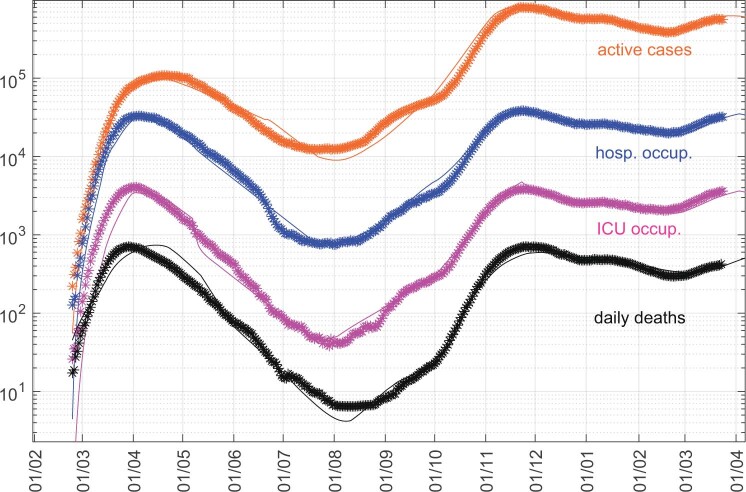
Epidemic evolution in Italy from late February 2020 to March 2021. Data (stars) and estimation (solid lines), based on the SIDARTHE model, of the time evolution of the epidemic, in logarithmic scale. Active cases (current diagnosed infected, related to the SIDARTHE variables R+T+D) are shown in orange; hospital occupancy (related to the SIDARTHE variables R+T) is shown in blue; ICU occupancy (related to variable T) is shown in magenta; daily deaths (related to the derivative of variable E) are shown in black.

The validation in Extended Data Fig. 1 shows how the SIDARTHE model (initially without vaccination) can faithfully reproduce the epidemic evolution observed so far. In the figures, the evolution over time of the number of active cases, hospitalizations and ICU occupancy, as well as daily deaths, is reported in logarithmic scale, comparing data (dots) with the model prediction (solid line). After 8 March 2020, a strict lockdown brought the Italian effective reproduction number below 1, successfully reversing the COVID-19 epidemic trend. Commercial and recreational activities gradually reopened, until the lockdown was fully lifted on 3 June. The lockdown relaxation coincided with a decreased risk perception and increased social gatherings when, after the first wave, restrictions were eased during the summer. Hence, as expected, a new upward trend in SARS-CoV-2 infections began in mid-August. The increase in daily cases, slow and steady at first, eventually led to a failure in the contact tracing system and the occurrence of a second wave. School reopening in the third week of September led to a steady increase in the number of new cases and hospital and ICU occupancy. This prompted the Italian government, on 4 November, to introduce a three-tier system enforcing diversely strict containment measures on a regional basis, depending on different risk scenarios. The slow decrease of the reproduction and hospitalization numbers led to stricter rules for the period of 24 December to 6 January. The initial onset of the second wave can be promptly identified at the beginning of August 2020, when the infection variables reached a minimum value. The descent phase of the second wave was much slower than that of the first, revealing that the enforced containment measures were milder. In particular, progressive countermeasures were enforced on 24 October (partial limitations), 4 November (regional lockdowns) and December (country-wide lockdown). Mild easing of restrictions and school reopening started on 7 January 2021, whereas other regional measures were implemented on 15 January, subsequently eased and then reinforced.

To reproduce the epidemic evolution over time, the system parameters are piecewise constant and are possibly updated at the following days:

[1 4 12 22 28 36 38 40 47 60 75 119 151 163 182 213 221 253 258 275 276 293 308 320 325 328 342 346 351 368 373 386]

The chosen parameter values are

*α* = [0.6588 0.4874 0.4886 0.3467 0.2311 0.2543 0.2543 0.3174 0.3467 0.3351 0.3236 0.3236 0.3467 0.4391 0.4045 0.3699 0.4869 0.4166 0.3567 0.3567 0.3267 0.3567 0.3932 0.3533 0.3533 0.4032 0.3034 0.3433 0.4471 0.4471 0.4471 0.3872]

*β* = [0.012 0.006 0.006 0.0053 0.0053 0.0053 0.0053 0.0053 0.0053 0.0053 0.0053 0.0053 0.0053 0.0053 0.0053 0.0053 0.0053 0.0053 0.0053 0.0053 0.0053 0.0053 0.0053 0.0053 0.0053 0.0053 0.0053 0.0053 0.0053 0.0053 0.0053 0.0053]

*γ* = [0.4514 0.2821 0.2821 0.1980 0.1089 0.1089 0.1089 0.1089 0.1089 0.1188 0.1188 0.1485 0.1485 0.1485 0.1485 0.1485 0.1485 0.1485 0.1485 0.1485 0.1485 0.1485 0.1485 0.1485 0.1485 0.1485 0.1485 0.1485 0.1485 0.1485 0.1485 0.1485]

*δ* = [0.0113 0.0056 0.0056 0.005 0.005 0.005 0.005 0.005 0.005 0.005 0.005 0.005 0.005 0.005 0.005 0.005 0.005 0.005 0.005 0.005 0.005 0.005 0.005 0.005 0.005 0.005 0.005 0.005 0.005 0.005 0.005 0.005]

*ε* = [0.1703 0.1703 0.1419 0.1419 0.1419 0.1419 0.1992 0.2988 0.2988 0.2988 0.2988 0.6972 0.2490 0.2988 0.2590 0.2294 0.3137 0.2868 0.2868 0.2988 0.2988 0.2988 0.2988 0.3988 0.3988 0.1988 0.2968 0.3088 0.2988 0.2988 0.2988 0.2988]

*θ* = [0.3705 0.3705 0.3705 0.3705 0.3705 0.3705 0.3705 0.5 0.5 0.5 0.5 0.5 0.5 0.6 0.3 0.6 0.37 0.370 0.37 0.37 0.37 0.37 0.37 0.37 0.37 0.37 0.37 0.37 0.37 0.37 0.37 0.37]

*ζ* = [0.1254 0.1254 0.1254 0.0340 0.0340 0.0341 0.0250 0.0250 0.0015 0 0.0001 0.0001 0.0005 0.0020 0.0030 0.0020 0.0046 0.0025 0.0025 0.0025 0.0025 0.0025 0.0025 0.0025 0.0025 0.0025 0.0025 0.0025 0.0025 0.0025 0.0025 0.0025]

*η* = [0.1054 0.1054 0.1054 0.0286 0.0286 0.0286 0.0286 0.021 0.0015 0 0 0 0.0005 0.002 0.0031 0.0026 0.003 0.0013 0.0013 0.001 0.0015 0.0018 0.0018 0.0018 0.0018 0.0018 0.0018 0.0018 0.0018 0.0018 0.0018 0.0018]

*μ* = [0.0205 0.0205 0.0205 0.0096 0.0084 0.0036 0.0036 0.0036 0 0 0 0.0036 0.0036 0 0.0024 0.0036 0.06 0.12 0.12 0. 0.12 0.12 0.12 0.12 0.12 0.12 0.12 0.12 0.12 0.12 0.12 0.12]

*ν* = [0.03 0.03 0.01 0.01 0.01 0.008 0.007 0.006 0.005 0.004 0.025 0.025 0.0026 0.0026 0.0026 0.002 0.002 0.002 0.002 0.02 0.02 0.02 0.02 0.02 0.02 0.02 0.02 0.02 0.02 0.02 0.02 0.02]

*τ*_2_ = [0 0 0 0 0 0 0.035 0.045 0.045 0.045 0.4500 0.45 0.02 0 0 0 0 0.0005 0.0005 0.17 0.17 0.17 0.17 0.17 0.17 0.17 0.17 0.17 0.17 0.17 0.17 0.17]

*τ*_1_ = [0.02 0.02 0.02 0.02 0.02 0.02 0.02 0.05 0.01 0.01 0 0 0 0 0 0 0.018 0.018 0.001 0.001 0.005 0.001 0.005 0.005 0.005 0.005 0.005 0.005 0.005 0.005 0.005 0.005]

*λ* = [0.0482 0.0482 0.0482 0.1128 0.1128 0.1128 0.1128 0.1128 0.1128 0.1128 0.1128 0.1128 0.1128 0.1128 0.1128 0.1128 0.1128 0.1128 0.1128 0.1128 0.1128 0.1128 0.1128 0.1128 0.1128 0.1128 0.1128 0.1128 0.1128 0.1128 0.1128 0.1128]

*ρ* = [0.0342 0.0342 0.0342 0.017 0.017 0.017 0.02 0.022 0.022 0.045 0.045 0.045 0.02 0.018 0.018 0.018 0.018 0.018 0.032 0.032 0.032 0.032 0.032 0.032 0.032 0.032 0.032 0.032 0.032 0.032 0.032 0.032]

*κ* = [0.0171 0.0171 0.0171 0.0171 0.0171 0.0171 0.02 0.022 0.022 0.035 0.035 0.035 0.02 0.02 0.02 0.02 0.02 0.02 0.02 0.02 0.02 0.02 0.02 0.02 0.02 0.02 0.02 0.02 0.02 0.02 0.02 0.02]

*χ* = [0.00025 0.00025 0.00025 0.00025 0.00025 0.00025 0.00025 0.0083 0.0083 0.0207 0.012 0.012 0.0037 0.0019 0.0019 0.00067 0.00067 0.00067 0.00015 0.022 0.022 0.032 0.022 0.0220 0.022 0.022 0.0120 0.022 0.012 0.012 0.012 0.012]

*σ* = [0.0513 0.0513 0.0513 0.0513 0.0513 0.0513 0.03 0.03 0.06 0.075 0.003 0 0 0 0.024 0.024 0.024 0.024 0.0024 0.024 0.024 0.024 0.024 0.024 0.024 0.024 0.024 0.024 0.024 0.024 0.024 0.024]

and lead to the following piecewise constant parametric reproduction number:

$${\cal{R}}_0$$ = [2.5200 1.7692 1.9725 1.3859 0.9593 1.0448 0.9162 0.8533 1.0138 0.8997 0.8715 0.5008 1.1494 1.3398 1.3776 1.4154 1.3439 1.2464 1.0104 0.9827 0.9097 0.9812 1.0685 0.9726 0.8150 0.9113 1.0709 0.9516 1.1747 1.2004 1.2651 1.0579]

Starting with day 405 (5 April 2021), the parameters are differentiated depending on the different scenarios associated with the presence of new virus variants and/or of the adoption of different restrictions:

- High transmission: *α* = 0.477, leading to a constant ${\cal{R}}_0 = 1.27$
- Open–Close: *α* switches every month between the high value 0.477 and the low value 0.3198; hence, every month ${\cal{R}}_0$ switches between the values 1.27 and 0.9, with an average value of about 1.1.
- Constant *α* = 0.4092, leading to ${\cal{R}}_0 = 1.1$
- Close–Open: *α* switches every month between the low value 0.3198 and the high value 0.477; hence, every month ${\cal{R}}_0$ switches between the values 0.9 and 1.27, with an average value of about 1.1. The Close–Open strategy features the same pattern as the Open–Close strategy, the only difference being that it starts with a closure phase.
- Eradication: *α* = 0.3198, leading to a constant ${\cal{R}}_0 = 0.9$.

Different parameter choices for the SIDARTHE-V model might yield the same ${\cal{R}}_0$. However, we have fitted our model based on a long history of data (since February 2020) and on a priori information about the epidemic and its management; to show the effect of a different ${\cal{R}}_0$, we are only modifying the infection parameters—that is, the high-sensitivity parameters (according to the thorough sensitivity analysis performed in our previous work^2^) that are affected by the spread of more transmissible variants and by NPIs. If two different combinations of parameters could fit equally well all the past data history and yield the same ${\cal{R}}_0$, the resulting long-term future evolution would also be similar. Any parameter choice that successfully reproduces the observed total number of cases is suitable for our goal: through the SIDARTHE-V model, we are computing only the predicted total number of detected infection cases, which is then projected onto healthcare system costs (including deaths and hospitalizations) by the data-based model.

The vaccination function is chosen according to the three different profiles shown in Extended Data Fig. 4, where $\varphi \left( {S\left( t \right)} \right) = \varphi \left( t \right)$ is piecewise constant (of course, $\varphi \left( {S\left( t \right)} \right) = 0$ when *S*(*t*) = 0).

In the adaptive vaccination scenarios in Extended Data Figs. 6 and 7, conversely, the vaccination function is chosen as ${\mathrm{max}}\{ \left[ {1 - r\left( {D\left( t \right) + R\left( t \right) + T\left( t \right)} \right)} \right]\varphi \left( t \right);0\}$, where *φ*(*t*) is the same piecewise constant vaccination profile as above, whereas the parameter *r* is chosen as *r* = 10^−6^.

Note that our model accounts for the effective immunization rate, regardless of the adopted vaccine. Any vaccine can be included in the model without altering its validity: the resulting immunization curves can be derived with the same procedure, regardless of the specific vaccine that has been used to achieve immunity.

**Extended Data Fig. 2:**
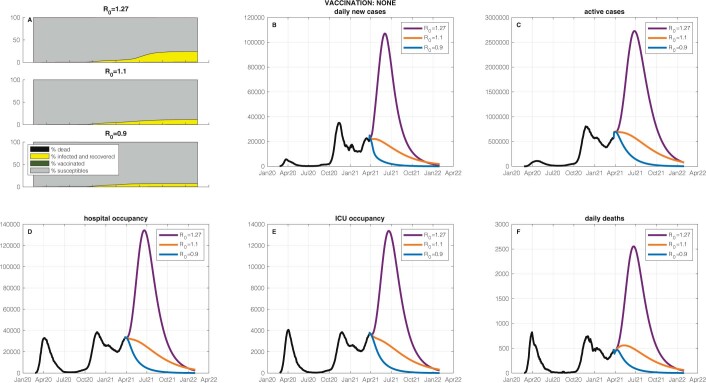
Epidemic evolution without vaccination for different constant values of ${\cal{R}}_0$. Time evolution of the epidemic, in the absence of vaccination, when different constant values of ${\cal{R}}_0$, namely ${\cal{R}}_0 = 1.27$ (purple), ${\cal{R}}_0 = 1.1$ (orange), ${\cal{R}}_0 = 0.9$ (blue), are assumed, resulting from different variants and/or containment strategies. **a**, Time evolution of the fractions of susceptibles, infected and recovered, and dead. **b,** Daily new cases. **c**, Active cases. **d**, Hospital occupancy. **e**, ICU occupancy. **f**, Daily deaths.

**Extended Data Fig. 3:**
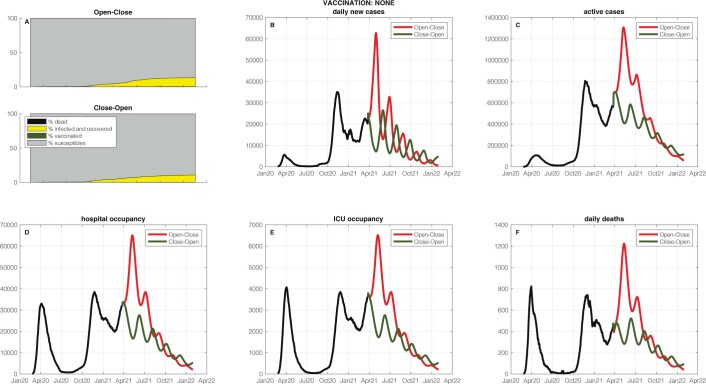
Epidemic evolution without vaccination for different intermittent strategies. Time evolution of the epidemic, in the absence of vaccination, when two alternative intermittent strategies are enforced, with an average value of ${\cal{R}}_0$ equal to 1.1. The Open-Close strategy (red) switches every month between ${\cal{R}}_0 = 1.27$ and ${\cal{R}}_0 = 0.9$, starting with ${\cal{R}}_0 = 1.27$. The Close-Open strategy (green) does the same, but starts with ${\cal{R}}_0 = 0.9$. **a**, Time evolution of the fractions of susceptibles, infected and recovered, and dead. **b**, Daily new cases. **c**, Active cases. **d**, Hospital occupancy. **e**, ICU occupancy. **f**, Daily deaths.

Adaptive vaccination under a medium vaccination schedule (Extended Data Figs. 6 and 7) leads to increased healthcare system costs with respect to piecewise constant vaccination functions (Figs. 2 and 3) for all ${\cal{R}}_0$ profiles. These costs can be compared with the even worse outcomes that are expected without vaccination, displayed in Extended Data Figs. 2 and 3. When ${\cal{R}}_0 = 1.27$, thousand deaths in the period from April 2021 to January 2022 increase from 90 to 229 (slow speed), from 72 to 220 (medium speed) and from 51 to 204 (fast speed), whereas they would be 298 without vaccination. Under the Open–Close strategy with average ${\cal{R}}_0$ equal to 1.1, thousand deaths in the period from April 2021 to January 2022 increase from 47 to 76 (slow speed), from 42 to 70 (medium speed) and from 35 to 62 (fast speed), whereas they would be 126 without vaccination. When ${\cal{R}}_0 = 1.1$, thousand deaths in the period from April 2021 to January 2022 increase from 34 to 48 (slow speed), from 30 to 44 (medium speed) and from 25 to 38 (fast speed), whereas they would be 96 without vaccination. Under the Close–Open strategy with average ${\cal{R}}_0$ equal to 1.1, thousand deaths in the period from April 2021 to January 2022 increase from 27 to 37 (slow speed), from 24 to 33 (medium speed) and from 21 to 28 (fast speed), whereas they would be 84 without vaccination.

### Data-driven model of healthcare system costs

To predict the evolution of deaths from the time series of reported cases, a field estimate of the apparent CFR is needed. This parameter is affected by the testing protocol, the healthcare system reaction and the age distribution of vaccinated people. For these reasons, a specific model should be derived for each country resorting to a data-based approach. We considered the Italian case, but the methodology has general validity and could be promptly applied to other countries. The input–output model predicting deaths from the new cases was derived in two steps. First, a data-based dynamic model for the unvaccinated population was estimated from data collected during the second wave. The static gain of this dynamic model coincides with the CFR for the unvaccinated population. In the second step, this gain was multiplied by a time-varying function that accounts for the lethality decrease consequent to progressive vaccination of older people.

The input of the dynamic model is the time series of new cases *n*(*t*), and the output is the time series of daily deaths *d*(*t*). Given the variations of testing protocols and the large number of unreported cases during the first wave, the model was estimated using second-wave data collected within a 110-d window ending on 7 February 2021. More recent data were not used because the base CFR model should not be affected by vaccinations, whose effect is suitably incorporated in a second step as detailed below. The model assumes that the deaths at day *t* depend on the past new cases according to the equation

$$d(t) = \mathop {\sum }\limits_{i = 0}^\infty w(i)n(t - i)$$

where the weight *w*(*i*) denotes the fraction of individuals who became infected at day *t*-*i* who eventually die at day *t*. The CFR for the unvaccinated population is then given by

$$CFR_0 = \mathop {\sum }\limits_{i = 0}^\infty w(i)$$

To estimate the weights, an exponential model with delay was assumed:

$$w(i) = \left\{ {\begin{array}{*{20}{c}} {0,} & {i < k} \\ {bf^{i - k},} & {i \ge k} \end{array}} \right.$$

where *k* is the delay and *f* and *b* are unknown parameters. Because both the new cases and the daily deaths exhibit an apparent weekly seasonality, the original series were replaced by their 7-d moving average. Parameters were estimated via least squares using the function oe.m of the MATLAB System Identification Toolbox^44^. The estimation of *f* and *b* was repeated for all delays *k* ranging from 0 to 15, and the delay *k* = 3, associated with the best sum of squares, was eventually selected. The estimated parameters and their percent coefficients of variation were:

$$f = 0.948,CV\left( \% \right) = 0.311$$

$$b = 0.00141,CV(\% ) = 5.36$$

Then, recalling the formula for the sum of the harmonic series, the second-wave CFR for the unvaccinated population is given by

$$CFR_0 = \mathop {\sum }\limits_{i = 0}^\infty w(i) = \frac{b}{{1 - f}} = 0.027$$

**Extended Data Fig. 4:**
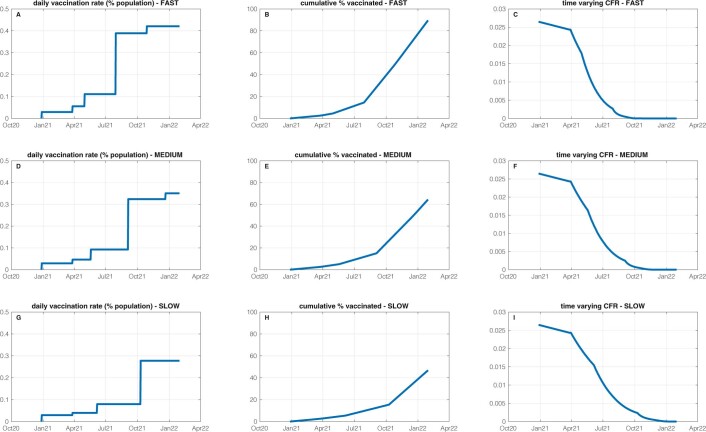
Effective vaccination schedules. Profiles of the considered three different effective vaccination schedules: slow, medium and fast. For each of the three schedules, we show: the evolution over time of the daily effective vaccination rate, namely the fraction of population successfully immunised (second dose) in one day (**a-d-g**), the cumulative fraction of immunised population as a function of time (**b-e-h**), the resulting time-varying Case Fatality Rate as a function of time, obtained taking population age classes into account and assuming a vaccination schedule that prioritises the elderly (**c-f-i**).

In the second step, the effect of vaccination on lethality was modeled by estimating a time-varying *CFR*(*t*) that depends on the vaccination schedule, described by the fraction *V*(*t*) of vaccine-immunized individuals at time *t*. Order of vaccination follows the reverse of the age. Slower or faster vaccination speeds correspond to different curves *V*(*t*), whose rate of increase might be less or more rapid. Three schedules were considered: fast, medium and slow. The fast schedule assumes that each the four phases, T1–T4, of the Italian vaccination plan^45^ is completed in one trimester. In the medium and slow schedule, the time was linearly extended by a factor 1.2 and 1.4, respectively. The three schedules are graphically displayed in Extended Data Fig. 4. To account for vaccines that were already administered, the scheduled vaccination rate from 27 December 2020 to 12 March 2021 was replaced by the actual average rate of second-dose vaccinations from 27 December to 19 February, considering that immunization develops with some weeks of delay after the administration.

**Extended Data Fig. 5:**
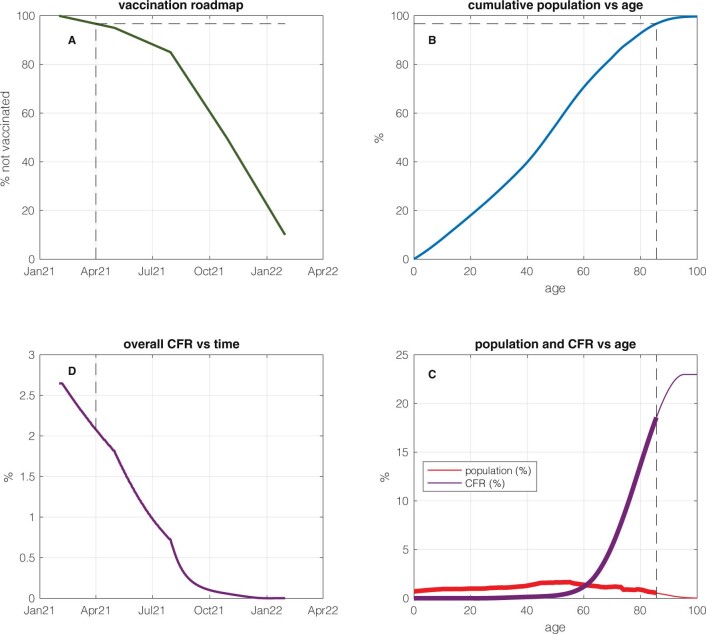
Time-varying age-dependent Case Fatality Rate as a function of the vaccination schedule. Computation of the time-varying Case Fatality Rate as a function of the vaccination roadmap, taking population age classes into account. **a**, Considered vaccination roadmap: fraction of non-vaccinated population over time. **b**, Cumulative Italian population as a function of age. **c**, Fraction of the population (red) and Case Fatality Rate (purple) as a function of age. **d**, Evolution of the overall Case Fatality Rate over time, assuming a vaccination schedule that gives priority to the elderly.

**Extended Data Fig. 6:**
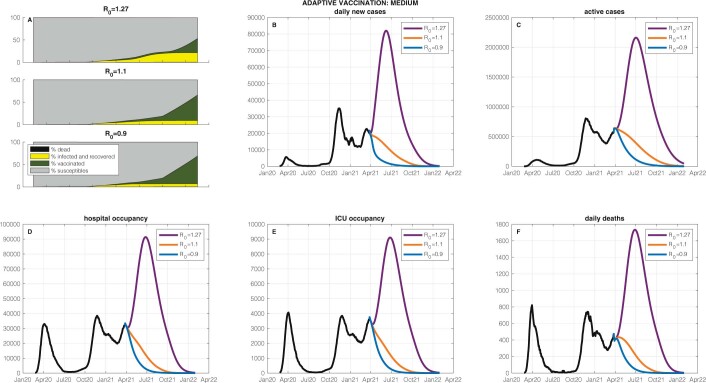
Epidemic evolution with adaptive vaccination for different constant values of ${\cal{R}}_0$. Time evolution of the epidemic, with adaptive vaccination, when different constant values of ${\cal{R}}_0$, namely ${\cal{R}}_0 = 1.27$ (purple), ${\cal{R}}_0 = 1.1$ (orange), ${\cal{R}}_0 = 0.9$ (blue), are assumed, associated with different variants and/or containment strategies. The adaptive vaccination speed is inversely proportional to the size of the epidemic, that is to the number of infection cases. **a**, Time evolution of the fractions of susceptibles, vaccinated, infected and recovered, and dead. **b**, Daily new cases. **c**, Active cases. **d**, Hospital occupancy. **e**, ICU occupancy. **f**, Daily deaths.

**Extended Data Fig. 7:**
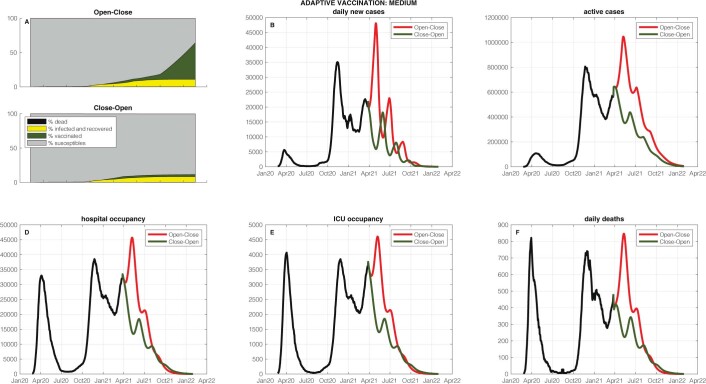
Epidemic evolution with adaptive vaccination for different intermittent strategies. Time evolution of the epidemic, with adaptive vaccination, when different intermittent strategies are enforced, with an average value of ${\cal{R}}_0$ equal to 1.1. The Open-Close strategy (red) switches every month between ${\cal{R}}_0 = 1.27$ and ${\cal{R}}_0 = 0.9$, starting with ${\cal{R}}_0 = 1.27$. The Close-Open strategy (green) does the same, but starts with ${\cal{R}}_0 = 0.9$. The adaptive vaccination speed is inversely proportional to the size of the epidemic, that is to the number of infection cases. **a**, Time evolution of the fractions of susceptibles, vaccinated, infected and recovered, and dead. **b**, Daily new cases. **c**, Active cases. **d**, Hospital occupancy. **e**, ICU occupancy. **f**, Daily deaths.

**Extended Data Fig. 8:**
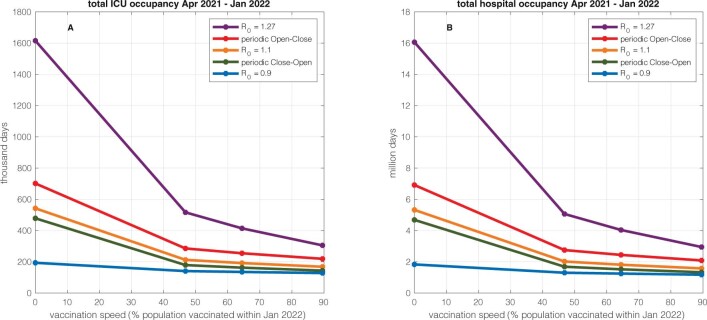
Health costs as a function of vaccination speed. Health-cost vs. vaccination-speed curves: for a given ${\cal{R}}_0$ profile, the curve gives the total ICU occupancy (**a**) and the total hospital occupancy (**b**) in the period from April 2021 to January 2022, as a function of the average vaccination speed, measured as the fraction of two-dose vaccinated population at the end of the period. The health-cost vs. vaccination-speed curves corresponding to a constant reproduction number are reported in purple (${\cal{R}}_0 = 1.27$), orange (${\cal{R}}_0 = 1.1$) and blue (${\cal{R}}_0 = 0.9$), while those corresponding to intermittent strategies are reported in red (Open-Close) and green (Close-Open).

The COVID-19 lethality *CFR*_*a*_ for an individual of age *a* was obtained by rescaling values published the Italian National Institute of Health^46^. Rescaling was necessary because the published values were inflated by the inclusion of patients who died during the first wave, when new cases were massively underreported. Rescaling was performed in such a way that the overall lethality coincides with *CFR*_0_ = 0.027. The profile *CFR*_*a*_ is displayed in Extended Data Fig. 5c.

If we assume that the number of deaths does not significantly affect the overall age distribution, the probability *P*(*Age* = *a*) can be directly inferred by ISTAT statistical tables^47^. The distribution was corrected by subtracting individuals who were already vaccinated on 19 February 2021, whose ages are made available on the vaccine open data repository^48^. The distribution of population by age is displayed in Extended Data Fig. 5c.

Recalling that *V*(*t*) is the fraction of vaccine-immunized individuals, and vaccination order follows the reverse of the age, the probability of death for an individual of age *a* who becomes infected at time *t* is

$$P(Death|Age = a,Infected,t) = \left\{ {\begin{array}{*{20}{c}} {0,} & {V(t) > P(Age > a)} \\ {CFR(a),} & {V(t) \le P(Age > a)} \end{array}} \right.$$

Then, the time-varying CFR is obtained by the total probability theorem:

$$CFR(t) = \mathop {\sum }\limits_{a = 0}^{100} P(Death|Age = a,Infected,t)P(Age = a|Infected,t)$$

where $P(Age = a|Infected,t)$ denotes the probability that the age of an individual is *a*, knowing that the individual became infected at time *t*. During the second wave, the age distribution of the infected individuals^46^ was similar to the age distribution of the Italian population. For instance, 56% of the population is in the age range 0–50 years, 28% is in the range 51–70 years and 16% is older than 70 years. In comparison, 55.6% of diagnosed cases between 18 December 2020 and 10 January 2021 were in age range 0–50 years, 28% in the range 51–70 years and 16.4% over 70 years. Therefore, $P(Age = a|Infected,t) = P(Age = a)$, so that the CFR of an individual infected at time *t* can be computed as

$$CFR(t) = \mathop {\sum }\limits_{a = 0}^{100} P(Death|Age = a,Infected,t)P(Age = a)$$

The steps of the procedure for the computation of *CFR*(*t*) are summarized in Extended Data Fig. 5. The time-varying profiles *CFR*(*t*) for the three vaccination schedules are plotted in Extended Data Fig. 4. Because protection against hospitalization and death has been observed already after the first dose, the calculation of the time-varying CFR was based on first dose administration, assumed twice as fast as second dose administration.

Finally, the input–output model that accounts for the effect of vaccination is given by

$$d(t) = \mathop {\sum }\limits_{i = 0}^\infty w(i)C(t - i)n(t - i),C(t) = \frac{{CFR(t)}}{{CFR_0}}$$

Due to the time-varying coefficient *C*(*t*), the same number of new cases will yield fewer and fewer deaths as vaccination comes to protect older segments of the population.

Dynamic models for hospital and ICU occupancies were developed in a similar way. The estimated parameters and their percent coefficients of variation for hospital occupancy (estimated delay *k* = 0) were:

$$a = 0.9522,CV\left( \% \right) = 0.226$$

$$f = 0.0694,CV(\% ) = 4.26$$

Those for ICU occupancy (estimated delay *k* = 0) were:

$$a = 0.953,CV\left( \% \right) = 0.328$$

$$f = 0.00677,CV(\% ) = 6.297$$

Assuming that gravity reduction parallels the lethality one, the effect of vaccination on hospital and ICU occupancies was described by modulating the input through the time-varying coefficient *C*(*t*).

**Extended Data Fig. 9:**
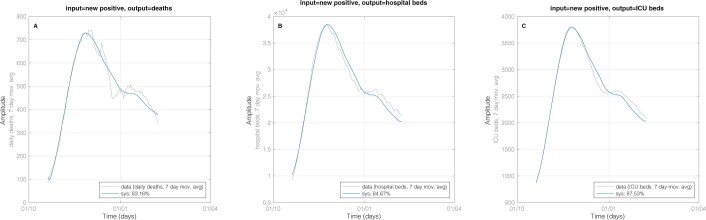
Dynamic input-output models for health costs. Estimation of the three dynamic input-output models linking new cases (input) to three outputs: deaths (**a**), hospital occupancy (**b**) and ICU occupancy (**c**). Each panel displays the observed outputs (grey) and the values predicted by the identified dynamic system (blue). In order to allow for weekly oscillations both input and output series were prefiltered with a 7-day moving average before performing nonlinear least squares fitting of a first-order model with delay. The FIT ratios of the three models are 83.16%, 84.67%, and 87.53%.

As seen in Extended Data Fig. 9, the three data-based dynamic models provide a very good fitting of deaths and hospital and ICU occupancies.

### Our scenarios: different values of ${\mathbf{{\cal{R}}}}_0$

The chosen values of ${\cal{R}}_0$ are based on plausible scenarios, in view of what has been observed throughout the past year in Italy, with a suitable combination of (1) presence of SARS-CoV-2 variants with increased transmissibility and (2) enforced restrictions. In particular, the mild restrictions that kept ${\cal{R}}_0$ around 0.9 with the original virus strain would keep it around 1.3 (at least) if the new variants increase transmissibility of at least 40–50%, as reported in the literature^49–51^. In fact, values around ${\cal{R}}_0$ = 1.3 were observed in mid-March 2021 in areas of Italy where the UK variant is becoming dominant. We have not considered even worse scenarios, because a higher ${\cal{R}}_0$ is unlikely to be sustainable: even stricter restrictions would then be enforced to prevent it from increasing. On the other hand, we deem it unlikely that restrictions so stringent to bring ${\cal{R}}_0$ below 0.9 would be enforced; hence, we have chosen this value as the other extreme scenario.

### Our scenarios: intermittent strategies, Open–Close and Close–Open

In some of our scenarios, we consider intermittent strategies that rely on the alternation of Open phases (associated with a larger ${\cal{R}}_0$) and Close phases (associated with a smaller ${\cal{R}}_0$) with a fixed proportion.

One of our main results is that, when planning over a fixed time period and having to set the order of two phases (Open and Close) with fixed length, starting with the Close phase is always an advantage. This happens because the associated healthcare system costs depend on the total number of infection cases in the considered time period, which is much larger if the Open phase comes first; hence, starting with a Close phase drastically reduces health costs and losses. On the other hand, socioeconomic costs are proportional to the duration and stringency of the restrictions, regardless of when they are enforced; hence, intermittent closures yield similar socioeconomic costs. Given a finite horizon and Close/Open phases of fixed length, closing before opening does not bring any additional socioeconomic cost, with respect to opening before closing, because the closing (and opening) phases have the same duration in both scenarios.

This principle holds true irrespective of the initial size of the epidemic. For instance, with very low case numbers, starting with a Close phase could approach or even achieve eradication, after which reopening would be completely safe (such an eradication approach has been successfully adopted, for example, by New Zealand^52,53^), whereas, if starting with an Open phase, the number of infection cases would grow exponentially, and the following Close phase could only mitigate the epidemic expansion. Of course, the higher the initial case number, the more visible the difference between the two strategies.

Also, the principle (if Open and Close phases with a fixed proportion need to be alternated in a periodic fashion, then starting with a Close phase is always preferable) remains valid regardless of the phase duration. In our scenarios, we have chosen 1-month-long phases, because this is a frequently observed choice in many countries (for example, in accordance with implemented policies in Italy but also in Israel) and is a sufficiently long time for the effect of interventions to be well visible.

### Reporting Summary

Further information on research design is available in the Nature Research Reporting Summary linked to this article.

## Online content

Any methods, additional references, Nature Research reporting summaries, source data, extended data, supplementary information, acknowledgements, peer review information; details of author contributions and competing interests; and statements of data and code availability are available at 10.1038/s41591-021-01334-5.

## Supplementary information

Reporting Summary

## Data Availability

We gathered all epidemiological and demographic data from publicly available sources: https://github.com/pcm-dpc/COVID-19/tree/master/dati-andamento-nazionale, https://www.epicentro.iss.it/coronavirus/bollettino/Bollettino-sorveglianza-integrata-COVID-19_13-gennaio-2021.pdf and http://dati.istat.it/Index.aspx. Data are also included in Extended Data Figs. 1 and 9 and in the code folder: https://giuliagiordano.dii.unitn.it/docs/papers/VaccineVariantsCode.zip.

## Code availability

The codes are available at https://giuliagiordano.dii.unitn.it/docs/papers/VaccineVariantsCode.zip.
